# Quantitative Study on MFL Signal of Pipeline Composite Defect Based on Improved Magnetic Charge Model

**DOI:** 10.3390/s21103412

**Published:** 2021-05-13

**Authors:** Bin Liu, Ning Luo, Gang Feng

**Affiliations:** College of Information Science and Engineering, Shenyang University of Technology, Shenyang 110870, China; liubinsgd@sut.edu.cn (B.L.); sgdxinxifg@163.com (G.F.)

**Keywords:** MFL internal detection, magnetic charge, composite defects, signal characteristic

## Abstract

Pipeline magnetic flux leakage (MFL) internal detection technology is the most widely used and effective method in the field of long-distance oil and gas pipeline online detection. With the improvement of data quantization precision, the influence of stress on MFL signal has been paid more and more attention. In this paper, the relationship between stress and saturation magnetization is introduced based on J-A theory. The analytical model of MFL detection signal for pipeline composite defects is established. The MFL signal characteristics of composite defects are quantitatively calculated. The effect of stress on MFL signal is studied. The theoretical analysis is verified by experimental data and excavation results. The researches show that the saturation magnetization of ferromagnets decreases exponentially with the increase of stress in strong magnetic field. The MFL signal of composite defect is weaker than that of volumetric defects of the same dimension. The axial amplitude and radial peak-to-peak value of MFL signal decrease with the increase of stress around the defect. The axial amplitude and radial peak-to-peak value of MFL signal increase non-linearly with the increase of width and depth of defects. When using MFL signal to judge the defect depth, it is necessary to make clear whether there is stress concentration phenomenon around the defect because the stress will lead to underestimation of the defect depth.

## 1. Introduction

Pipeline transportation plays an important role in the transportation of oil and natural gas. It is of great economic and social significance to ensure the essential safety of oil and gas pipelines. At present, pipeline internal detection technology is internationally recognized as the most effective means of oil and gas pipeline safety maintenance [1,2]. The detector which pushed by the pipeline conveying medium can detect the corrosion, metal loss, crack, stress and other damage on the pipeline wall dynamically without stopping production and transmission [3,4]. Traditional pipeline internal detection techniques, such as magnetic leakage internal detection, electromagnetic ultrasonic internal detection, magnetic memory internal detection, and so on, play an important role in field of online detection of different types of pipeline defects [5,6,7].The MFL detection technology has become the mainstream technology of pipeline detection in the world because of its advantages, such as strong anti-interference ability, fast signal acquisition speed, and no need of coupling agent [8,9].

Due to the influence of the internal pressure, the stress around the defect of the pipeline in service is far greater than the average value of the pipeline stress. Some of the stress concentrated in areas can accumulate to a certain extent, which will form micro cracks. The composite defects caused by stress-assisted corrosion have macroscopic defects and stress damage simultaneously [10]. Many scholars have studied the relationship between pipe stress and magnetic signal, mostly using magnetic memory measurement (MMM) technology [11,12]. The magnetic memory method is used to detect materials in the geomagnetic environment without external excitation magnetic field [13]. The detected magnetic signal is the self-magnetic flux leakage (SFML) signal of the material [14,15]. Compared with traditional MFL detection signals, the MMM signals are unstable and easy to disturb, so the MMM technology has not been widely used in the online internal detection of long-distance oil and gas pipelines. In order to adapt to the widely used technology of MFL internal detection, some scholars have studied the magnetic signals of defects with stress under excitation state [16]. The magnetic signal characteristics of composite defects under near saturation magnetization were obtained through experiment and simulation. It is known from experiment that the MFL signal of defect is affected by the stress. The traditional mathematical model of MFL internal detection ignores the influence of stress on magnetic signal and has errors in the analysis of the dimension and damage degree of composite pipe defects. Therefore, it is necessary to establish a model to calculate the MFL signal of composite defects in saturated magnetization state. It is helpful to study the influence of stress variation on MFL signals. In this paper, the relationship between stress and magnetization of ferromagnetic materials is studied. A numerical model of composite defect in pipelines for internal detection is established based on the magnetic charge model introducing the stress operator. The characteristics of MFL signals of composite defects with different dimensions are quantitatively calculated. The effects of stress intensity and stress gradient on MFL signals of composite defects are analyzed. The systematic experiment is carried out and the project excavation results is analyzed. The research lays a foundation for quantitative analysis of composite defects in the future.

## 2. Numerical Model of Composite Defect

### 2.1. Classic Model

The classical analytical formula for MFL is [17]:

(1) $$d\vec{H}=\frac{\rho_{s}dS}{4\pi \mu_{0}|r{|}^{3}}\vec{r},$$

where ***H*** is the magnetic field intensity, *ρ_s_* is the magnetic charge density of defect side wall, *dS* is the area element of magnetic charge surface, ***r*** is the direction vector from the magnetic charge surface to the detection position, *r* is the distance, and *μ_0_* is the vacuum permeability.

In this paper, the defect is on the large-diameter long distance oil and gas pipelines. The pipelines with an outer diameter of 1219 mm are usually made of X80 steel. Due to the large diameter, the surface radian of the pipeline can be regarded as a plane.

**Figure 1:**
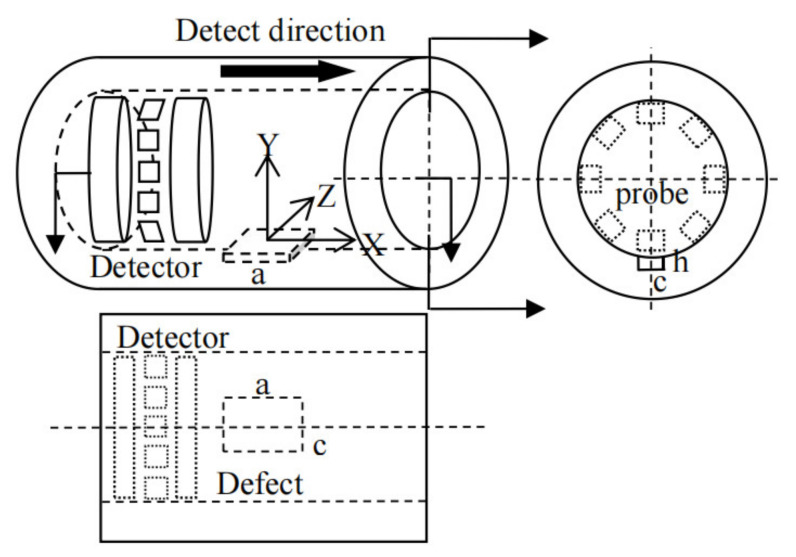
Schematic diagram of MFL internal detection model.

As shown in Figure 1, the Cartesian coordinate system is established on the inner surface of the pipeline. The axial direction of the pipeline is defined as the *X* axis, the circumferential axis is defined as the *Z* axis, the radial axis is defined as the *Y* axis. During the internal detection of axial MFL, the detector moves along the axial direction. The magnetic field intensity at the probe is obtained by integral of Equation (1):

(2) $$\vec{H}=\int_{-y}^{0}\int_{z_{1}}^{z_{2}}\int_{x_{1}}^{x_{2}}\frac{\rho_{s}dS}{4\pi \mu_{0}|r{|}^{3}}\vec{r}dxdydz,$$

where, (*x_1_,x_2_*) is the axial interval of magnetic charge stacking surface, *y* is the depth of magnetic charge stacking surface, and (*z_1_*,*z_2_*) is the circumferential interval of magnetic charge stacking surface.

Assuming that the depth of unstressed volumetric type defect is *h*, the length is *a* and the width is *c*. The defect is on the inner surface of the pipe. The magnetic charges are piled on both sides of axial defect cross-sections. When the detector is in position (*x_0_*, *y_0_*, *z_0_*), the MFL signal of defect detected along the X direction can be obtained as:

(3) $$\left\{ \begin{array}{ll} H_{x}=\frac{\mu_{s}M_{s}}{4\pi \mu_{0}}\{\int_{-h}^{0}\int_{-\frac{c}{2}}^{\frac{c}{2}}\frac{(x_{0}+a/2)dydz}{{\left[{\left(x_{0}+a/2\right)}^{2}+{\left(y_{0}-y\right)}^{2}+{\left(z_{0}-z\right)}_{}^{2}\right]}^{\frac{3}{2}}} & \\ +\int_{-h}^{0}\int_{-\frac{c}{2}}^{\frac{c}{2}}\frac{(x_{0}-a/2)dydz}{{\left[{\left(x_{0}-a/2\right)}^{2}+{\left(y_{0}-y\right)}^{2}+{\left(z_{0}-z\right)}_{}^{2}\right]}^{\frac{3}{2}}}\} & y\in (-h,0)\\ H_{y}=\frac{\mu_{s}M_{s}}{4\pi \mu_{0}}\{\int_{-h}^{0}\int_{-\frac{c}{2}}^{\frac{c}{2}}\frac{(y_{0}-y)dydz}{{\left[{\left(x_{0}+a/2\right)}^{2}+{\left(y_{0}-y\right)}^{2}+{\left(z_{0}-z\right)}_{}^{2}\right]}^{\frac{3}{2}}} & z\in (-\frac{c}{2},\frac{c}{2})\\ +\int_{-h}^{0}\int_{-\frac{c}{2}}^{\frac{c}{2}}\frac{(y_{0}-y)dydz}{{\left[{\left(x_{0}-a/2\right)}^{2}+{\left(y_{0}-y\right)}^{2}+{\left(z_{0}-z\right)}_{}^{2}\right]}^{\frac{3}{2}}}\} & \end{array}\right.$$

where ***M_s_*** is the saturation magnetization of pipeline material and *μ_s_* is the relative permeability of a material at saturation magnetization.

### 2.2. Improved Model

#### 2.2.1. Stress Operator

Assuming that the length of the stress concentration region at both ends of the defect is *b* along the X direction. According to the stress distribution of the defect under load, the stress at the axial section of the defect is the largest, and gradually decreases to the average stress level of the pipeline with the distance from the end face of the defect [18]. Considering the variation of magnetic properties of materials caused by the stress concentration phenomenon, the MFL detected signal is the leakage magnetic field of stress region and defect. The stress operator should be introduced into the classical magnetic charge model.

According to the J-A model, the magnetization of the material can be expressed as [19]:

(4) $$M=cM_{an}+(1-c)M_{irr},$$

where ***M*** is the magnetization, ***M_irr_*** is the irreversible component of the magnetization, *c* is the reversible coefficient, and ***M_an_*** is the non-hysteresis magnetization. The non-hysteresis magnetization can be expressed as [20]:

(5) $$M_{an}=M_{s}[\coth(\frac{H_{eff}}{a}-\frac{a}{H_{eff}})],$$

where ***M_s_*** is the saturation magnetization, *a* is a constant, and ***H_eff_*** is the equivalent magnetic field of the material which can be expressed as [21]:

(6) $$H_{eff}=H+H_{\sigma }+\alpha M,$$

where *α* is a coupling parameter, ***H*** is the external magnetic field, and ***H_σ_*** is the equivalent magnetic field generated by the stress which can be expressed by the empirical formula of magnetostriction coefficient and magnetization [22]:

(7) $$H_{\sigma }=\frac{3}{2}\frac{\sigma }{\mu_{0}}{\left(\frac{d\lambda }{dM}\right)}_{\sigma }=\frac{3\sigma }{\mu_{0}}\sum_{i=0}^{\infty }i\gamma_{i}(\sigma )M^{2i-1}=\frac{3\sigma }{\mu_{0}}\sum_{i=0}^{\infty }\left(iM^{2i-1}\sum_{n=0}^{\infty }\frac{\sigma^{n}}{n!}\gamma_{i}^{n}(0)\right),$$

where *γ_i_^n^*(0) is the nth derivative of *γ_i_* with respect to stress at *σ* = 0.

The derivative of the external magnetic field of (4) is shown in (8), where the irreversible component of the magnetization can be expressed as (9) [23]:

(8) $$\frac{dM}{dH}=c\frac{dM_{an}}{dH}+(1-c)\frac{dM_{irr}}{dH},$$

(9) $$M=M_{an}-\frac{k\delta }{\mu_{0}}\frac{dM_{irr}}{dH},$$

where *k* is the nailing coefficient of the material, *δ* = ±1. The calculation formula of magnetization curve with stress operator is:

(10) $$\frac{dM}{dH}=\frac{-\frac{\mu_{0}}{k\delta }(M_{an}-M)-\frac{c}{1-c}\frac{dM_{an}}{dH}}{\frac{\mu_{0}}{k\delta }(M_{an}-M)[\frac{3\sigma }{\mu_{0}}(\gamma_{1}(\sigma )+6M^{2}\gamma_{2}(\sigma ))]-\frac{c}{1-c}},$$

**Figure 2:**
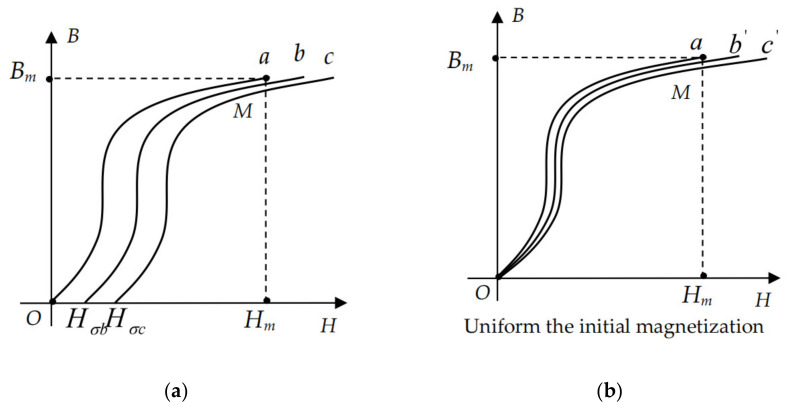
Influence diagram of stress on magnetic flux intensity: (**a**) The magnetization curve which the stress can not affect magnetization intensity; (**b**) The magnetization curve which the stress affect magnetization intensity.

The magnetization curve of ferromagnetic materials without stress is shown in Figure 2a, Curve a. Assuming that the stress does not change the magnetization of ferromagnetic materials, the stress equivalent field is only used as an additional external magnetic field. The magnetization curve of the material is shown in Figure 2a, Curves b and c. According to Equation (6), the equivalent field which is the initial additional field increases with the stress increases, so the stress of Curve c is greater than that of Curve b. In the actual measurement, the stress equivalent additional magnetic field is not visible, which is reflected by the variation of magnetization intensity. As shown in Figure 2b, the initial magnetization intensity is the same, and Curves b’ and c’ represent the magnetization curves of materials under different stresses. Under the same magnetic field intensity *H_m_*, the magnetic flux intensity of ferromagnets decreases with the increase of stress.

**Figure 3:**
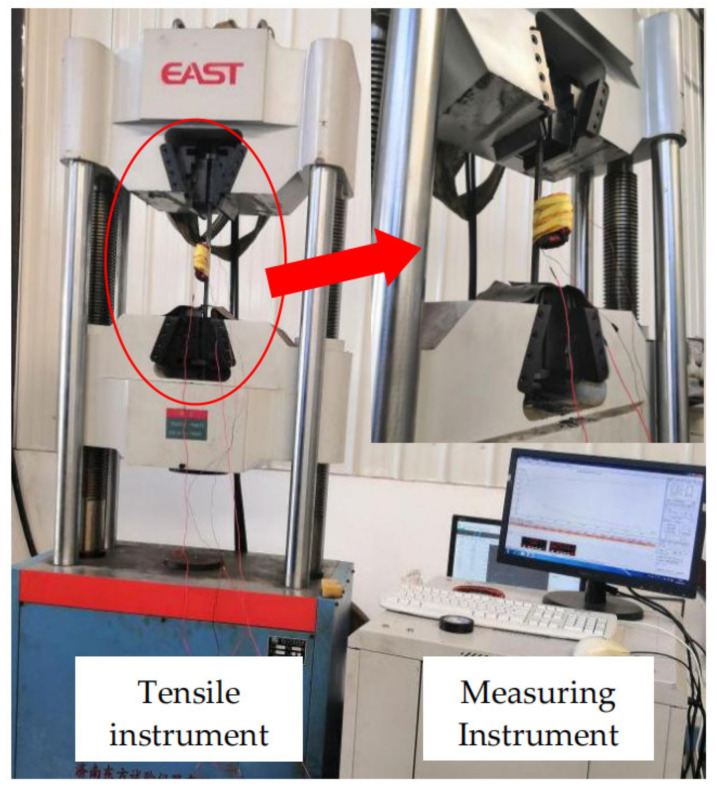
Magnetization curve measurement.

According to the above principle, the magnetic parameter measuring instrument is used to measure the magnetization curves of X80 steel under different stresses. The instrument is shown in Figure 3. The measuring coil is fixed on the specimen. The length of specimen is 450 mm, the width is 10 mm and the thickness is 10 mm. The specimen is stretched by tensile machine, and the stresses ranging from 20 MPa to 200 MPa are applied to the specimens by adjusting the tensile force.

**Figure 4:**
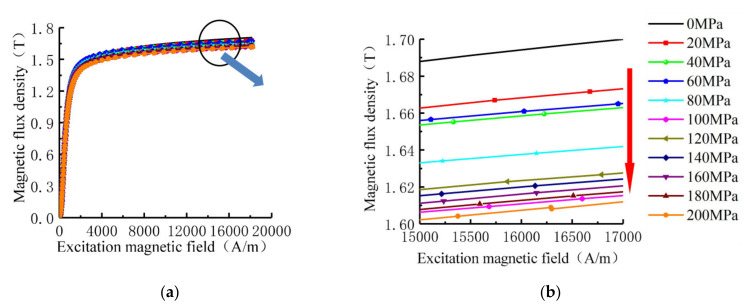
Magnetization curves of X80 steel under different stresses: (**a**) Complete magnetization curve; (**b**) Near saturated region curve.

The magnetization curves of the measured specimens under different stresses are shown in Figure 4.

As shown in Figure 4. The stress affects the magnetization curve of X80 steel. In the nearly saturated region where the external magnetic field is 15 kA/m to 17 k/m, the magnetic flux intensity of the material weakens with the increases of the stress, which is the same as the magnetic flux intensity under *H_m_* in the theoretical analysis of Figure 2.

Based on the measured B-H curve, Equation (11) is used to calculate the saturation magnetization of X80 steel tested under different stresses.

(11) $$B_{s}=\mu_{0}(H_{0}+M_{s}),$$

where *B_s_* is the measured saturation magnetic flux intensity, *H_0_* is the external magnetic field strength of steel, and *M_s_* is the saturation magnetization. The saturated external magnetic field intensity is set as 18 kA/m, and the variation trend of experimental data under different stresses can be fitted.

**Figure 5:**
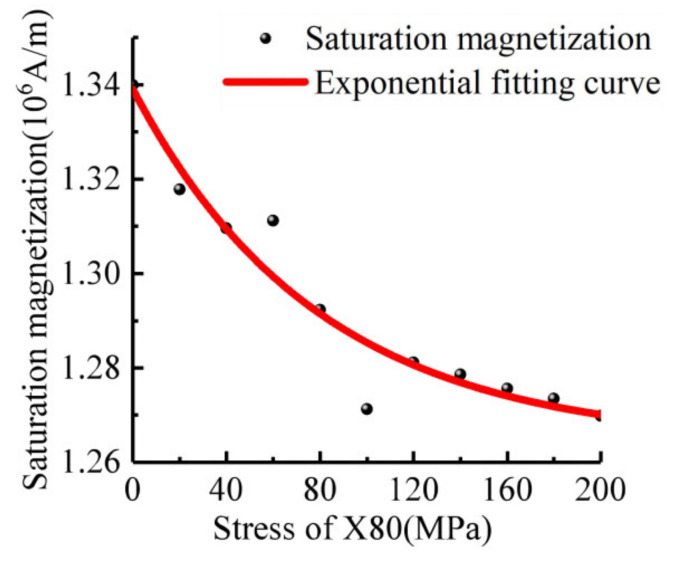
X80 Curve of saturation magnetization with stress.

As shown in Figure 5, the saturation magnetization decreases exponentially with the increases of stress, and the fitting formula is:

(12) $$M_{s}=1.264+0.075\times {\left(0.9875\right)}^{\sigma },$$

where the unit of *σ* in the Equation is MPa, and the unit of *M_s_* is 10^6^ A/m. Equation (12) is the stress operator in the calculation of magnetic flux leakage, reflecting the quantitative relationship between stress and magnetic parameters of materials, which can be introduced into the calculation model of composite defects.

#### 2.2.2. Application of Stress Operator

**Figure 6:**
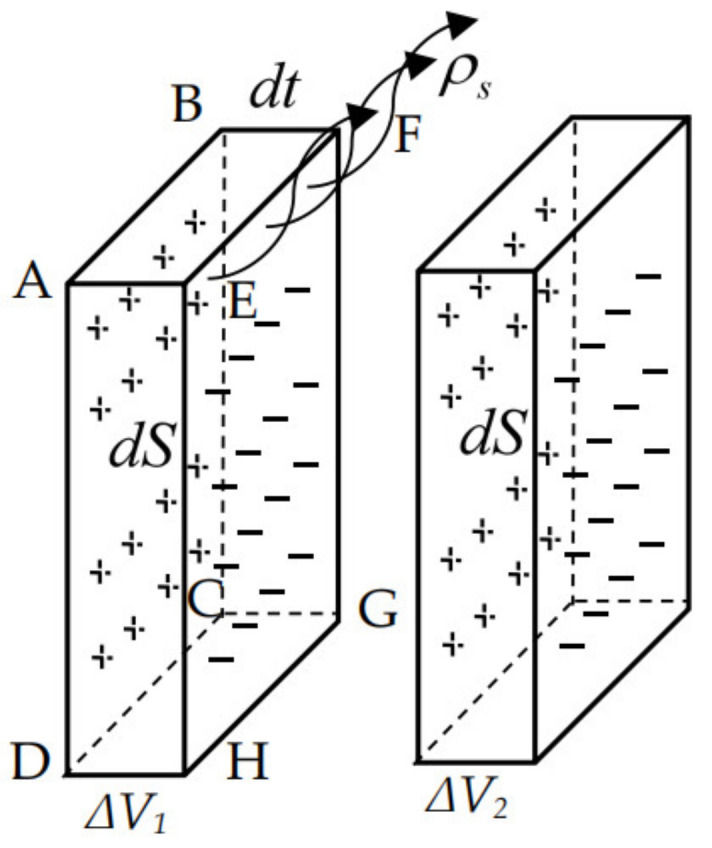
Schematic diagram of magnetic charge density calculation in stress region.

The ferromagnet in the magnetic field produces a dipole moment along the direction of the magnetic field, which produces the polarization phenomenon of bound magnetic charge on the surface of the ferromagnet. The continuously varying stress region is regarded as a collection of finite continuous volumetric units Δ*V*, and the magnetic polarity intensity ***J*** is the vector sum of magnetic dipole moments in unit volumetrice.

(13) $$J=\frac{\sum P_{m}}{\Delta V},$$

where ***P_m_*** is the magnetic dipole moment. As shown in Figure 6, assuming that the left surface (ABCD) of Δ*V* has a bound magnetic charge +*ρ*, the right surface (EFGH) of Δ*V* has a bound magnetic charge -*ρ*. The magnetic moment of Δ*V* is *m* = *ρdSdt*. From the definition of polarization intensity, the magnetic dipole moment and the magnetic moment represent the same physical reality.

(14) $$J=\frac{\rho dSdt}{\Delta V_{1}}=\rho ,$$

As shown in Equation (14). The magnetic charge density on the surface of the unit is numerically equal to its magnetic polarity intensity. The relationship between magnetic polarization intensity and magnetization intensity is *J* = *μ_0_ M*, the magnetic charge density is *ρ* = *μ_0_ M*. Assuming that Δ*V_1_* and Δ*V_2_* are two adjacent stress volumetric units in Figure 6. The magnetization of Δ*V_1_* is *M_1_*, the magnetization of Δ*V_2_* is *M_2_*, the magnetic charge is *ρ_1_* and *ρ_2_* respectively. According to the polarity of magnetic charge, a stacked magnetic charge *ρ_s_* is generated at the intersection of the two volume units.

(15) $$\rho_{s}=\mu_{0}(M_{1}-M_{2}),$$

Assuming that the stress in the stress concentration area varies uniformly along the axial direction. The maximum stress is *σ_max_*. The average pipe stress is *σ_0_*. The relationship between stress and axial position *x* is shown in Equation (16). (*x_1_*, *x_2_*) is the axial range of the stress concentration area.

(16) $$\sigma (x)=\left\{\begin{array}{c} \frac{2x(\sigma_{\max}-\sigma_{0})}{\left(x_{2}-x_{1}\right)}+\;\sigma_{0}\;\;,x\in \left(x_{1},\frac{x_{2}-x_{1}}{2}\right)\\ -\frac{2x(\sigma_{\max}-\sigma_{0})\;}{\left(x_{2}-x_{1}\right)}+\sigma_{\max},x\in \left(\frac{x_{2}-x_{1}}{2},x_{2}\right) \end{array}\right.,$$

By substituting Equations (12) and (16) into (15), the relationship between magnetic charge density and stress at each position can be obtained as:

(17) $$\rho_{x}\;\;\;\;=0.075\mu_{0}\times {\left(0.9875\right)}^{\sigma (x)}\times [{\left(0.9875\right)}^{2\Delta x\frac{\sigma_{\max}-\sigma_{0}}{x_{2}-x_{1}}}-1],$$

where, the Δ*x* represents the axial thickness of the finite element. When Δ*x* approaching zero, the free magnetic charge density at each point in the axial position of the stress concentration region can be obtained. The parameters *f_x_* = (0.9875)*^σ^*^(*x*)^ represents the variation trend of saturation magnetization of the material, which is affected by position, stress gradient and maximum stress. The Δ*x* affects the accuracy of analytical calculation.

Assuming that the center of the surface of the stress region is the origin of the coordinate, the axial length of the stress region is *b*, the circumferential width is *c*, and the depth is *h*. The intensity of the MFL signal at the spatial point (*x_0_*, *y_0_*, *z_0_*) is:

(18) $$\left\{\begin{array}{ll} H_{x}=\int_{-\frac{b}{2}}^{\frac{b}{2}}\int_{-\frac{c}{2}}^{\frac{c}{2}}\frac{\rho_{x}h}{4\pi \mu_{0}}f_{x}dxdz & x\in \;(-\frac{b}{2},\frac{b}{2})\\ H_{y}=\int_{-\frac{b}{2}}^{\frac{b}{2}}\int_{-\frac{c}{2}}^{\frac{c}{2}}\frac{\rho_{x}h}{4\pi \mu_{0}}f_{y}dxdz & z\in (-\frac{c}{2},\frac{c}{2}) \end{array}\right.,$$

where, *f_x_* = (*x_0_*-*x*)/[(*x_0_*-*x*)^2^ + *y_0_^2^* + (*z*-*z_0_*)^2^]^3/2^ is the position parameters of the tangential signal in the stress region, *f_y_* = *y_0_*/[(*x_0_*-*x*)^2^ + *y_0_^2^* + (*z*-*z_0_*)^2^]^3/2^ is the position parameters of the normal signal in the stress region.

Introducing the stress operator into the classical MFL model can improve the computational accuracy of the model. Assuming that the length of the defect on the inner surface of the pipeline is *a*, the width is *c*, the depth is *h*. The length of stress concentration regions at both sides of defect is *b*. The improved MFL analytical formula for defects in the stress region is:

(19) $$\left\{ \begin{array}{ll} H_{x}=\frac{M_{\sigma \max}}{4\pi \mu_{\sigma \max}}\{\int_{-h}^{0}\int_{-\frac{c}{2}}^{\frac{c}{2}}\frac{(x_{0}+a/2)dydz}{{\left[{\left(x_{0}+a/2\right)}^{2}+{\left(y_{0}-y\right)}^{2}+{\left(z_{0}-z\right)}_{}^{2}\right]}^{\frac{3}{2}}} & \\ +\int_{-h}^{0}\int_{-\frac{c}{2}}^{\frac{c}{2}}\frac{\left(x_{0}-a/2\right)dx_{m}dc_{m}dh_{m}}{{\left[{\left(x_{0}-a/2\right)}^{2}+{\left(y_{0}-y\right)}^{2}+{\left(z_{0}-z\right)}_{}^{2}\right]}^{\frac{3}{2}}}\} & \\ +\frac{h}{4\pi }(\int_{-a-b}^{-a}\int_{-\frac{c}{2}}^{\frac{c}{2}}\rho_{x}f_{x}dxdz+\int_{a}^{a+b}\int_{-\frac{c}{2}}^{\frac{c}{2}}\rho_{x}f_{x}dxdz) & x\in (-b-\frac{a}{2},b+\frac{a}{2})\\ H_{y}=\frac{M_{\sigma \max}}{4\pi \mu_{\sigma \max}}\{\int_{-h}^{0}\int_{-\frac{c}{2}}^{\frac{c}{2}}\frac{(y_{0}-y)dydz}{{\left[{\left(x_{0}+a/2\right)}^{2}+{\left(y_{0}-y\right)}^{2}+{\left(z_{0}-z\right)}_{}^{2}\right]}^{\frac{3}{2}}} & y\in (-h,0)\;z\in (-\frac{c}{2},\frac{c}{2})\\ +\int_{-h}^{0}\int_{-\frac{c}{2}}^{\frac{c}{2}}\frac{(y_{0}-y)dydz}{{\left[{\left(x_{0}-a/2\right)}^{2}+{\left(y_{0}-y\right)}^{2}+{\left(z_{0}-z\right)}_{}^{2}\right]}^{\frac{3}{2}}} & \\ +\frac{h}{4\pi }(\int_{-a-b}^{-a}\int_{-\frac{c}{2}}^{\frac{c}{2}}\rho_{x}f_{y}dxdz+\int_{a}^{a+b}\int_{-\frac{c}{2}}^{\frac{c}{2}}\rho_{x}f_{y}dxdz) & \end{array}\right.$$

where, *M_σ_*_max_ is the saturation magnetization of pipe at the end of defect, *μ_σ_*_max_ is the relative permeability of the material corresponds to the magnetic intensity *M_σ_*_max_. Using Equation (19) and the stress operator of X80 steel, the signal of composite defects on X80 pipeline can be quantitatively calculated.

## 3. Analytical Calculation and Analysis

**Figure 7:**
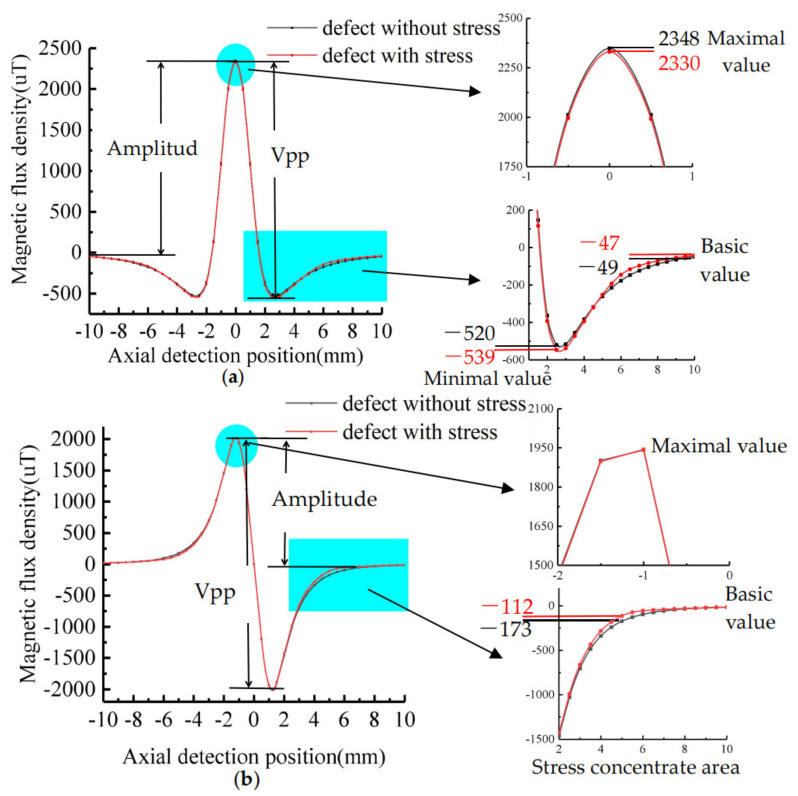
Comparison of quantitative calculation of MFL: (**a**) Tangential signal; (**b**) Normal signal.

The MFL signals of the same dimension defects are calculated by using the classical MFL analytical model and the improved MFL analytical model respectively. The classical model is in the state of no stress and the improved model is in the state with stress. Assuming that the pipe inner surface defect dimension is: *h* = 2 mm, c = 4 mm, a = 2 mm, b = 5 mm, σ_max_ = 200 MPa, the average stress of pipe is σ_a_ = 100 MPa. When the external magnetic field intensity is 18 kA/m, *M_σ_*_max_ = 1.27 × 10^6^ A/m, *μ_σ_*_max_ = 8.89 × 10^−5^ H/m, *M_a_* = 1.34 × 10^6^ A/m, *μ_a_* = 9.41 × 10^−5^ H/m. The tangential and normal components of MFL signal are obtained according to Equations (3) and (19). The signals are shown in Figure 7.

As shown in Figure 7, the characteristic strength of defect signal with stress is less than that of defect without stress. Since the signal characteristics of the stress-containing region and stress-free region in the defect are identical, only numerical differences exist, it is impossible to distinguish the existence of stress at the defect by signal characteristics. Compared with the MFL signal of defect without stress, the maximum value of tangential signal with stress decreases by 18 μT, the base value increases by 2 μT, the amplitude decreases by 20 μT, and the peak-to-peak value (vpp) has no variation. The maximum value and minimum value of the normal signal decrease by 10 μT, the base value decreases by 1μT, the amplitude decreases by 9 μT, and the peak-to-peak value decreases by 20 μT. The signal 5 mm away from the defect center increases by 61 μT. The intensity of MFL signal of each parameter is shown in Table 1 and Table 2.

It can be seen from the table that the MFL signal strength after introducing the stress operator is less than that calculated by the classical model, with an axial amplitude error of 0.83% and a radial peak-to-peak error of 0.52%. The following analysis is performed to investigate how the error is affected.

### 3.1. Influence of Defect Dimension on Signal

When using the magnetic charge model for quantitative calculation, it is necessary to consider the influence of the defect dimension on the magnetic charge. The magnetic charge of the volumetric defect is accumulated on the cross-section of the axial direction, which is influenced by the depth and width of the defect and independent of the length of the defect. This paper analyzes the influence of the depth and width on the MFL signal by introducing the stress operator.

#### 3.1.1. Depth Affects the Signal

**Figure 8:**
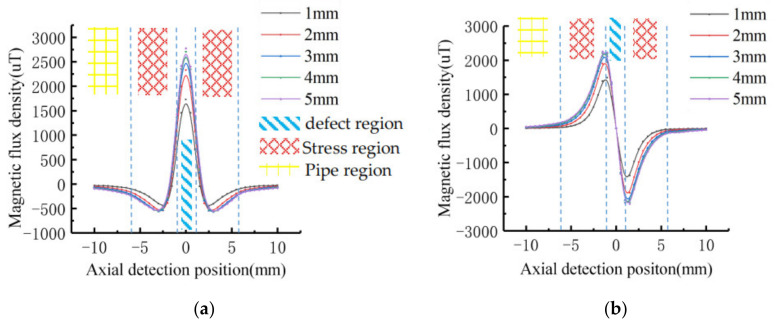
The MFL signals with stress operators at different depths: (**a**) Tangential signal; (**b**) Normal signal.

Assuming that the stress on the defect is constant. The dimension of defect is: *c* = 4 mm, *a* = 2 mm, *h* = 1 mm, 2 mm, 3 mm, 4 mm, 5 mm. The signal after introducing the stress operator is shown in Figure 8.

**Figure 9:**
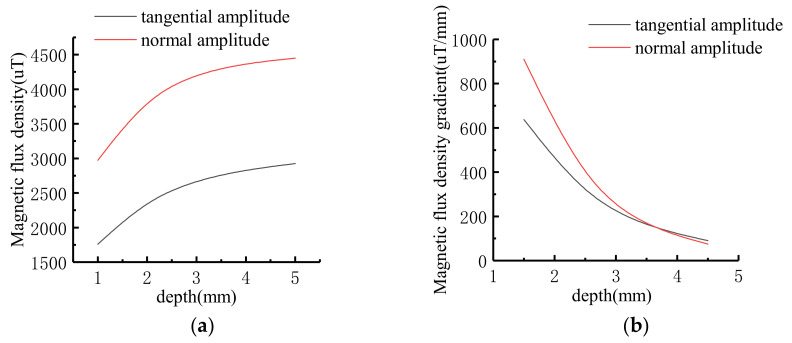
Variation trend of signal characteristics at different depths: (**a**) Signal variation curve; (**b**) Signal gradient variation curve.

The characteristic values of MFL signals at different depths were extracted, and the variation trend was shown in Figure 9. With the increase of defect depth, the amplitude of tangential component and the peak-to-peak value of normal component increase non-linearly. The signal variation gradient decreases as the defect depth increases.

**Figure 10:**
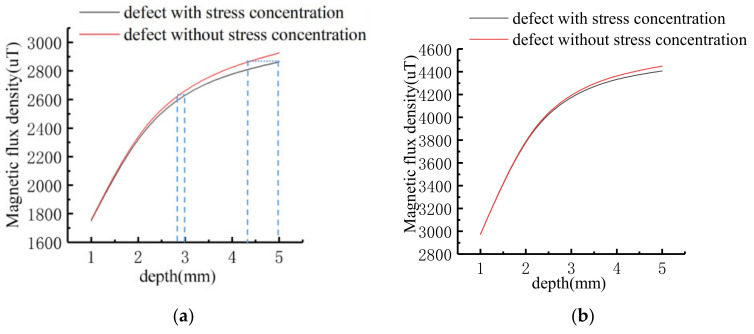
Comparison diagram of signal characters at different depths: (**a**) Tangential amplitude signal; (**b**) Normal peak-to-peak signal.

The signal characters of the stress-containing operator model and the classical model at different depths are compared. The result is shown in Figure 10.

As shown in Figure 10, the error between the calculated value of the improved model and the classical model gradually increases with depth. When the depth of defect is calculated from 1 mm to 5 mm, the tangential amplitude errors are 0.45%, 0.83%, 1.23%, 1.69%, 2.12%, and the normal peak-to-peak value errors are 0.2%, 0.52%, 0.9%, 1.28%, 1.62%, respectively. It can be seen that if the influence of stress is ignored when analyzing the data, the deep defect with stress will be judged as the shallow defect without stress. As shown in the tangential variation diagram, the amplitude of the tangential signal of 5 mm depth defect with stress is the same as 4.3 mm depth defect without stress. The amplitude of tangential signal of 3 mm -depth defect with the same stress is the same as that 2.7 mm depth defect without stress. The error of depth estimation caused by stress influence becomes larger with the increase of defect depth.

#### 3.1.2. Width Affects the Signal

**Figure 11:**
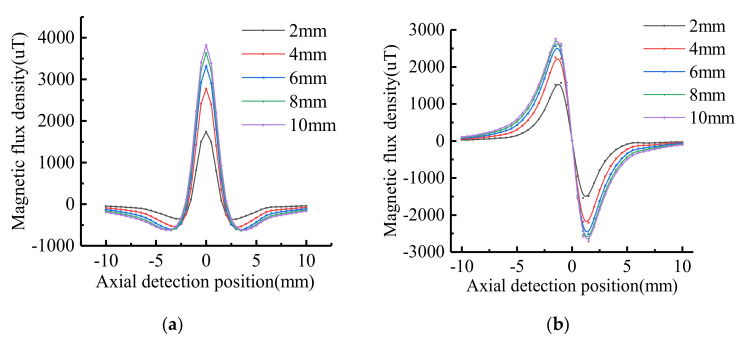
The MFL signals with stress operators at different widths: (**a**) Tangential signal; (**b**) Normal signal.

Assuming that the stress on the defect is constant. The dimension of defect is: *h* = 5 mm, *a* = 2 mm, *c* = 2 mm,4 mm,6 mm,8 mm,10 mm, respectively. The signal calculation after introducing the stress operator is shown in Figure 11.

**Figure 12:**
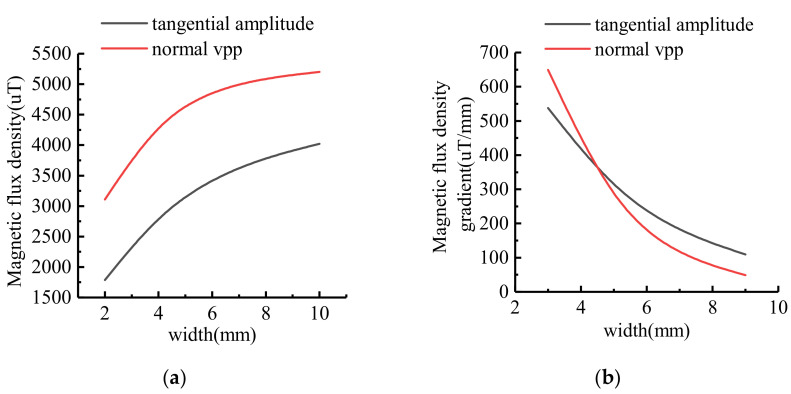
The variation trend of signal characteristics at different widths: (**a**) Signal variation curve; (**b**) Signal gradient variation curve.

The characteristic values of MFL signals with different widths were extracted, and the variation trend was shown in Figure 12. With the increase of defect width, the amplitude of tangential component and the peak-to-peak value of normal component increase non-linearly. The signal variation gradient decreases as the defect width increases.

**Figure 13:**
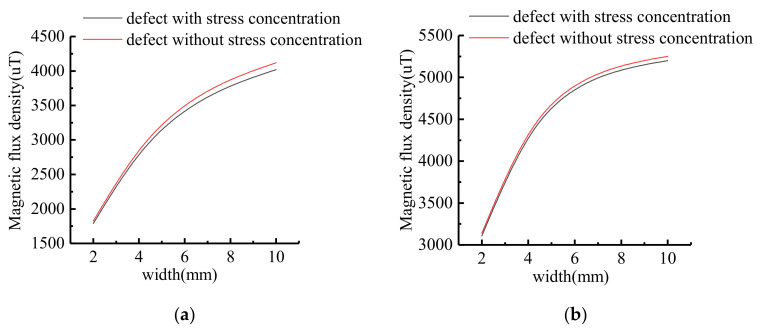
Comparison diagram of signal characters at different widths: (**a**) Tangential amplitude signal; (**b**) Normal peak-to-peak signal.

The signal characters of the stress-containing operator model and the classical model at different widths are compared. The result is shown in Figure 13.

As shown in Figure 13, the tangential amplitude errors are 1.94%, 2.11%, 2.26%, 2.36% and 2.43% respectively when calculating the defect with depth from 2 mm to 10 mm. The calculated values of the improved model and the classical model gradually increase with the width. The normal peak-to-peak value errors are both 0.95%, and the errors do not increase with the width.

### 3.2. Influence of Stress Density on Signal

**Figure 14:**
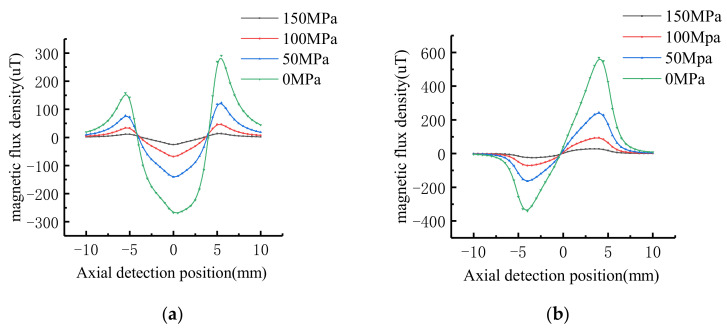
Signal characteristics of stress region with different stress gradient: (**a**) Tangential signal; (**b**) Normal signal.

In order to study the effect of stress gradient in stress region on MFL signal. The signal of the stress concentrate region is calculated by the improved model. The dimension of stress region is: *h* = 5 mm, *c* = 4 mm, *b* = 5 mm. The maximum stress in the stress region is *σ*_max_ = 200 MPa, the average stress of pipe is *σ_a_* = 150 MPa, 100 MPa, 50 MPa, 0 MPa respectively. The stress gradient can be calculated as 10 MPa/mm, 20 MPa/mm, 30 MPa/mm, 40 MPa/mm respectively. According to Equation (18), the MFL signal in the stress region is shown in Figure 14.

When the stress gradient is 40 MPa/mm, 30 MPa/mm, 20 MPa/mm and 10 MPa/mm respectively, the tangential signal amplitude is 311 μT, 160 μT, 75 μT, and 28 μT, respectively. The peak-to-peak values of normal signals is 832 μT, 388 μT, 163 μT, and 52 μT, respectively.

Similarly, the MFL signal can be calculated when the average stress of the pipeline is 0 MPa and the maximum stress is 50 MPa, 100 MPa, 150 MPa and 200 MPa respectively. The stress gradient can be calculated as 10 MPa/mm, 20 MPa/mm, 30 MPa/mm, 40 MPa/mm respectively. The tangential signal amplitude is 28 μT, 75 μT, 150 μT, and 200 μT, respectively. The peak-to-peak values of normal signals is 52 μT, 163 μT, 388 μT, and 832 μT, respectively.

It can be seen from the data that the characteristic intensity of MFL signal when the average stress of the pipeline is 0 MPa and the maximum stress is 50 MPa is consistent with that when the average stress area is 150 MPa and the maximum stress is 200 MPa. Therefore, the characteristic intensity of MFL signal is not affected by the pipeline pressure, but is related to the stress variation gradient in the stress region. The value of the characteristic variation of the MFL signal increases with the gradient of stress variation increases.

**Figure 15:**
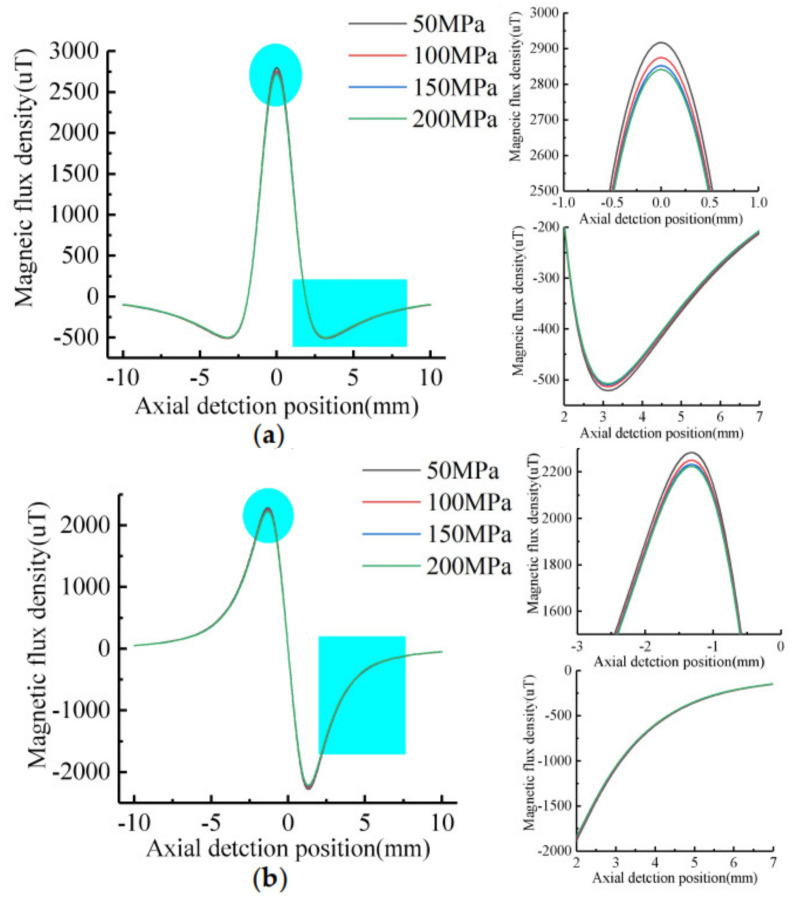
Signal characteristics of defects with different stress: (**a**) Tangential signal; (**b**) Normal signal.

In order to study the influence of the stress strength around the defect on the MFL signal, the dimension of the defect on the inner surface of the pipe is taken as: *h* = 5 mm, *c* = 4 mm, *a* = 2 mm. The axial length of stress region is *b* = 5 mm. The maximum stress is *σ*_max_ = 200 MPa, 150 MPa, 100 MPa, 50 MPa respectively. The stress gradient in the pipe stress region is 10 MPa/mm. The characteristics of the MFL signal of defect are shown in Figure 15.

As shown in Figure 15, the tangential signal maximum value decreases with the stress increases, the minimal value increases with the stress increases, and the normal extreme value decreases with the stress increases. Therefore, the stress maximum value affects the tangential amplitude of the leakage signal and the normal peak-to-peak value. The signal characteristics value decrease with the stress increases. Since the stress gradient value is constant, the signal of the stress region has no obvious variation.

**Figure 16:**
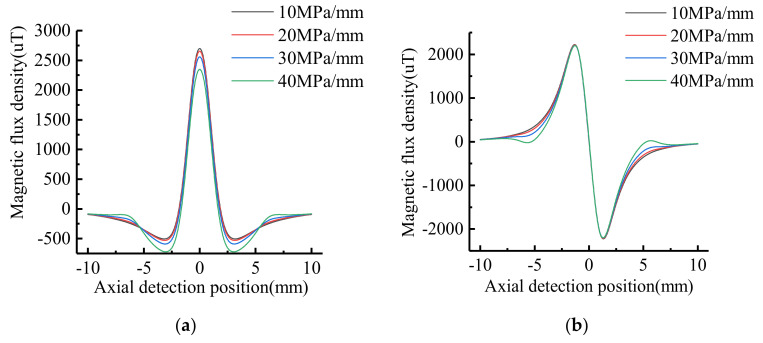
Signal characteristics of defects with different stress gradient: (**a**) Tangential signal; (**b**) Normal signal.

In order to study the influence of the stress gradient on the MFL signal, the dimension of the defect on the inner surface of the pipe is taken as: *h* = 5 mm, *c* = 4 mm, *a* = 2 mm.The axial length of stress region is *b* = 5 mm.The maximum stress is *σ*_max_ = 200 MPa. The stress gradient is 10 MPa/mm, 20 MPa/mm, 30 MPa/mm, 40 MPa/mm, respectively. The characteristics of the MFL signal of defect are shown in Figure 16.

As shown in Figure 16, the maximum and minimum values of tangential signal decrease with the stress gradient increases, but the amplitude remains the same. The normal extremes do not vary with the stress gradient, so the normal peak-to-peak value is constant. The signal gradient of tangential and normal component in the stress regions increase with the increase of stress gradient.

To sum up, the MFL signal of the defect is affected by the variation of stress around it. An increase in stress intensity leads to a weakening of the MFL signal in the defect region, and an increase in stress gradient leads to an increase in the gradient of the MFL signal in the stress region around the defect.

Based on the fatigue damage mechanism of the material and previous accident analysis, it is clear that simple volumetric defects and stress concentrations are not a concern, but rather that pipeline safety personnel are more concerned with volumetric defects containing stress. The analysis of quantitative calculations shows that the greater the stress concentration at the defect, the more likely it is to cause damage to the pipe. The error of the defect depth judgement without taking into account the effect of stress when analyzing the MFL signal resulting in the misdetection of pipeline safety personnel. Therefore, it is extremely important that the presence and intensity of stress at the defect location is clearly identified when analyzing the MFL signal of volumetric defect.

## 4. Experiment

**Figure 17:**
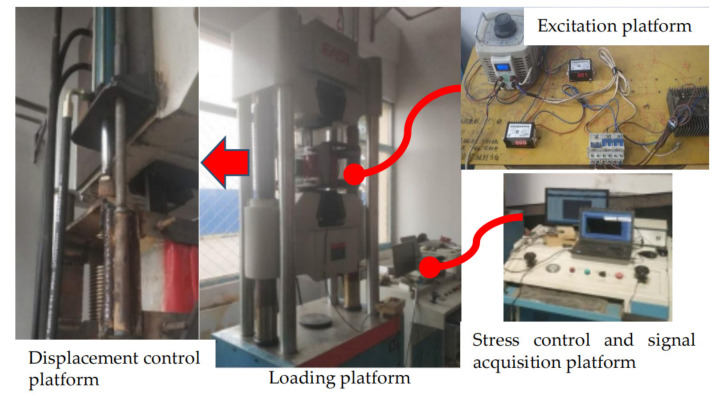
Experimental platform.

In order to verify the correctness of the theoretical model, a steel bar stress tension test was designed in this paper. The experimental device is shown in Figure 17.

As shown in Figure 17, the experimental platform is made up of four parts, including tensile loading device, displacement control device, excitation device and signal acquisition device. The tensile loading device is used to clamp specimens and impose uniform tension to the specimens. The displacement control device is used to fix the excitation coil and move at a constant speed. The excitation device is composed of DC coil, bridge circuit, AC transformers, voltmeter and ammeter. The transformer changes the excitation magnetic intensity by adjusting the voltage. The voltmeter and ammeter are used to observe excitation condition. The signal acquisition device is composed of a magnetic flux leakage probe fixed at the center of the coil and a host computer. The precision and resolution of the probe are the same as in engineering applications.

**Figure 18:**
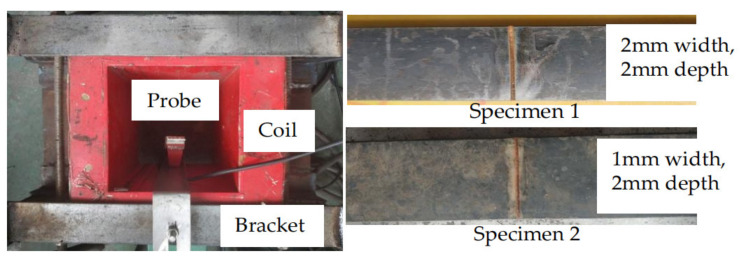
Excitation device and specimens.

The specimen is placed on the pulling machine and the coil is placed around the specimen. The coil current is controlled by the excitation platform to generate external magnetic fields of different strengths outside the specimen. The direction of the magnetic field is parallel to the direction of the tension. The hydraulic device is used to control the coil to move along the tensile direction of the steel bar. The probe passes directly above the defect to measure the MFL signal of the defect under different tensile forces. The specimen and probe are shown in Figure 18.

It has been calculated that an external magnetic field of 18 kA/m is generated at the probe when 5 A current is applied to the coil. Specimen dimensions are 700 mm long, 60 mm wide and 16 mm thick. For a 1 mm wide and 2 mm deep defect on the specimen, a tension of 80 kN applied by the tensile machine will produce a stress of 205 MPa at the end of the defect. For a 2 mm wide and 2 mm deep defect on the specimen, a tension of 80 kN will produce a stress of 162 MPa at the end of the defect. Because the average stresses in the specimens are all 83 MPa, the gradient of stress change was greater in specimen 1 than in specimen 2. In the experiments, a tensile force of 0 kN and 80 kN was applied to the two specimens and the defect leakage signals were measured at 18 kA/m respectively. The width and depth of defect in specimen 1 were consistent with the theoretical model, and the defect characteristics of specimen 2 ensured that the stress state was consistent with the theoretical model. The accuracy of the theoretical analysis was further verified by tensile tests of the two specimens.

**Figure 19:**
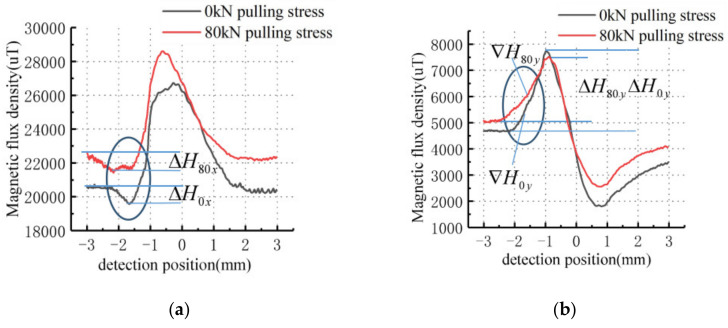
The MFL signals of specimen 1 under different stresses: (**a**) Tangential signal; (**b**) Normal signal.

The measured magnetic leakage data for Specimen 1 is shown in Figure 19.

As shown in Figure 19, the horizontal coordinate of the signal is the distance detected and the vertical coordinate is the intensity of the detected magnetic leakage signal. The tangential amplitude of the leakage signal without stress is approximately 6500 μT with a base value of approximately 20,500 μT and the normal peak-to-peak value is approximately 5750 μT with a base value of approximately 4500 μT. The characteristic intensity of the defective leakage signal is in error with the theoretical calculation, and is larger than the theoretical calculation for the same depth. The reason for this is that the stress zone width in the theoretical calculation is 4 mm and the crack width in the experiment is 60 mm to ensure that the tensile stress at the edge of the defect is the same. From the base value of the tangential signal, the magnetic field strength above the specimen is approximately 16.32 kA/m, which is weaker compared to the theoretically calculated excitation.

When the specimen is pulled at 80 kN, the tangential signal amplitude is approximately 6200 μT with a base value of approximately 21,500 μT and the normal signal peak value is approximately 5000 μT with a base value of approximately 5000 μT. The contrast shows that the characteristic intensity of the defect leakage signal under stress is weakened. Due to the stress concentration phenomenon at the defect end, the gradient of the tangential leakage signal above the stress region is less than that in the unstressed region (ᐁ*H_80 y_* < ᐁ*H_0 y_*). The differential between the maximum peak value and the base value of the tangential leakage signal with stress defect is less than that in the unstressed region (Δ*H_80 y_* < Δ*H_0 y_*). The differential between the minimum value and the base value above the stress region of the normal signal with stress defect is greater than that in the unstressed region (Δ*H_80 x_* > Δ*H_0 x_*), as marked in the Figure 19.

**Figure 20:**
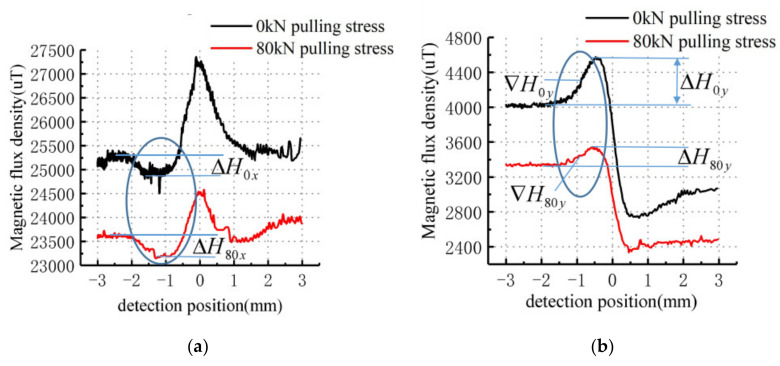
The MFL signals of specimen 2 under different stresses: (**a**) Tangential signal; (**b**) Normal signal.

To verify the repeatability of the phenomenon, specimen 2 was tested and its leakage signal is shown in Figure 20.

As shown in Figure 20, the tangential amplitude of the leakage signal without stress is approximately 2250 μT with a base value of approximately 25,000 μT and the normal peak-to-peak value is approximately 1900 μT with a base value of approximately 4000 μT. When the specimen is pulled at 80 kN, the tangential signal amplitude is approximately 1250 μT with a base value of approximately 23,500 μT and the normal signal peak value is approximately 1100 μT with a base value of approximately 3400 μT. Compared to 0 kN tension, the tangential characteristic strength of specimen 2 is reduced by 1000 μT, which is greater than 300 μT for specimen 1. The normal characteristic strength of specimen 2 is reduced by 800 μT, which is greater than 750 μT for specimen 1. This result is consistent with the theoretical analysis that the greater the gradient of the stress region, the greater the weakening of the leakage signal.

By comparing the measured data with the calculated results of the model, the tangential amplitude and normal peak-to-peak value of the MFL signal are affected by the stress at the defect edge. The values are smaller than the eigenvalues without stress. The decrease amplitude measured by the test is larger than that calculated by the model, which is due to the larger defect width during the test. The accuracy of the theoretical analysis was verified by tensile testing of the above specimens.

## 5. Project Verification

To further verify the phenomenon of theoretical analysis, magnetic flux leakage internal detection and excavation verification were carried out for a long-distance natural gas transmission pipeline with a diameter of 1219 mm, and the wall thickness of the pipeline was 18.4 mm.

**Figure 21:**
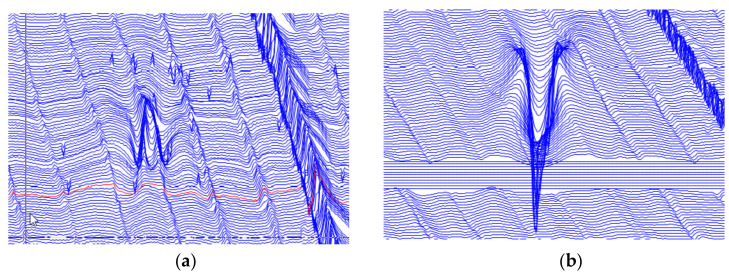
The MFL signals in different years: (**a**) Tangential component of first year MFL signal; (**b**) Tangential component of second year MFL signal.

A volumetric defect in the outer wall of the pipe was selected for verification by reading the MFL internal detection data of the pipeline. The MFL detection characteristics of the defect are shown in Figure 21.

Compare Figure 21 with Figure 20a. Because the excitation magnetic fields of the first year and the second year are opposite, the signal characteristics of the two tests are in opposite directions. The characteristics of engineering testing are the same as those of experiment. There is a peak value at the defect of tangential signal and trough values on both sides of the defect. The differential between the maximum peak value and the base value of the tangential leakage signal in Figure 21a is less than that in b. The differential between the minimum trough value and the base value above the stress region of the tangential signal in Figure 21a is greater than that in b. The phenomenon means the stress of defect in Figure 21a is larger than that in b and the depth of defect in Figure 21a is smaller than that in b according to the result of experiment.

**Figure 22:**
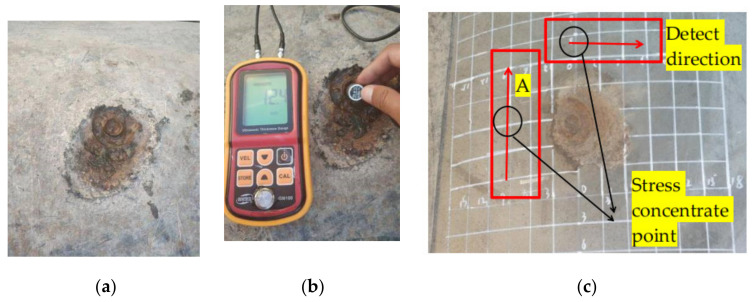
The excavation verification results: (**a**) Damage of anticorrosion coating; (**b**) Ultrasonic thickness measurement; (**c**) Pipe wall damage.

Based on data analysis, the volumetric defect was determined to be 8% of the wall thickness of the pipe at the first inspection, which was approximately 1.5 mm depth. The defect was judged as a common volumetric defect without stress influenced. When the defect was detected the following year, this volumetric defect was determined to be 28% of the wall thickness of the pipe at a depth of approximately 5 mm. According to the channel distribution of the detector, there was no significant change in the defect range. Due to the short interval between two detection times and the large expansion of defect depth, special attention was needed. Therefore, excavation verification was carried out in the same year. The excavation verification results are shown in Figure 22.

**Figure 23:**
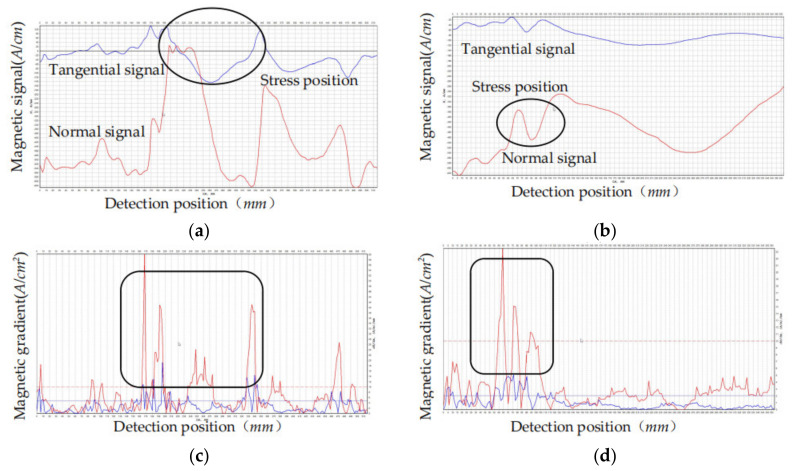
The magnetic memory measurement results: (**a**) Detection signal in region A; (**b**) Detection signal in region B; (**c**) Signal gradient in region A; (**d**) Signal gradient in region B.

As can be seen from the ultrasonic thickness measurement, the wall thickness of the deepest position of defect is 12.4 mm and the depth of the defect reaches 33% of the wall thickness, approximately 6 mm, which is higher than the analytical value of the internal inspection data. The magnetic memory was measured in regions A and B in Figure 22, respectively, and the results are shown in Figure 23.

As marked in Figure 23a,b,the changes of magnetic memory signal were detected in both region A and region B. According to Figure 23c,d,the magnetic signal gradient is larger than the other position. The stress concentration phenomenon was detected in region A and region B judging by the gradient value of the magnetic memory data. Compared with Figure 22c, the stress concentration phenomenon was detected around the defect. As a result, the stress region around the defect affected the judgment of the MFL signal, which was consistent with the theoretical analysis.

## 6. Conclusions

Magnetic flux leakage (MFL) internal detection technology is the main detection method for safety assessment of long-distance oil and gas pipelines. The traditional quantitative model of MFL detection neglects the influence of stress on saturation magnetization signal, which leads to errors in the analysis results. In this paper, by introducing the stress operator into the magnetic charge model, a mathematical model for the internal detection of composite pipe defects was established, and the influence of stress on the MFL signal was studied. The test data were used to verify the results, and the following conclusions were drawn: The saturation magnetization of ferromagnets decreases exponentially with the increase of stress in strong magnetic field. The MFL signal of composite defect is weaker than that of volumetric defects of the same dimension. The axial amplitude and radial peak-to-peak value of MFL signal decrease with the increase of stress around the defect. The degree of stress concentration around the defect does not affect the axial amplitude and radial peak-to-peak value, but the gradient of MFL signal in the stress area around the defect increases with the increase of the stress concentration degree. The axial amplitude and radial peak-to-peak value of MFL signal increase non-linearly with the increase of width and depth of defects, and the signal enhancement gradient decreases with the increase of depth and width. The differential between the maximum peak value and the base value above the defect region of the tangential leakage signal increases with the depth of defect increases. The differential between the maximum peak value and the base value above the defect region of the tangential leakage signal decreases with the stress of defect increases. The differential between the minimum trough value and the base value above the stress region of the tangential signal increases with the stress of defect increases. The stress will lead to underestimation of the defect depth.

## Figures and Tables

**Table 1:** Characteristic strength of tangential signal.

Defect Types	Tangential Signal(μT)				
	Max	Min	Base	Amplitude	Vpp
With no stress	2348	−520	−47	2397	2868
With stress	2330	−539	−49	2377	2869

**Table 2:** Characteristic strength of normal signal.

Defect Types	Normal Signal(μt)			
	5 mm Away from the Center	Absolute Value of Base	Absolute Value of Extremum	Amplitude
With no stress	173	15	1941	1926
With stress	112	14	1931	1917
